# An Overview of Cognitive Radio Technology and Its Applications in Civil Aviation

**DOI:** 10.3390/s23136125

**Published:** 2023-07-03

**Authors:** Ruikang Zheng, Xuan Li, Yudong Chen

**Affiliations:** 1 China Airport Planning & Design Institute Co., Ltd. Northwest Branch, Xi’an 710075, China; zhengrk@cacc.com.cn (R.Z.); xbchenyd@cacc.com.cn (Y.C.); 2 School of Information Engineering, East China Jiaotong University, Nanchang 333000, China

**Keywords:** cognitive radio, applications and trends, challenges, civil aviation

## Abstract

This paper provides an overview of cognitive radio technology and its applications in the field of civil aviation. Cognitive radio technology is a relatively new and emerging field that allows for dynamic spectrum access and efficient use of spectrum resources. In the context of civil aviation, cognitive radio technology has the potential to enable more efficient use of the limited radio spectrum available for communication and navigation purposes. This paper examines the current state of cognitive radio technology, including ongoing research and development efforts, regulatory issues, and potential challenges to widespread adoption. The potential applications of cognitive radio technology in civil aviation are also explored, including improved spectrum utilization, increased safety and security, and enhanced situational awareness. Finally, the paper concludes with a discussion of future research directions and the potential impact of cognitive radio technology on the future of civil aviation. It is hoped that this paper will serve as a useful resource for researchers, engineers, and policy makers interested in the emerging field of cognitive radio technology and its potential applications in the field of civil aviation.

## 1. Introduction

Since the concept of cognitive radio (CR) was proposed by Joseph Mitola III in 1998, it has attracted lots of attention in scientific research organizations and institutions. In recent years, due to the increasing shortage of radio spectrum resources, CR technology came into being. Through the perception and analysis of radio spectrum, cognitive radio can access the idle spectrum resource opportunity for data transmission, improve spectrum utilization, and reduce spectrum waste. Therefore, cognitive radio technology is widely used in military, commercial, and civil aviation, and other fields.

However, with the continuous development of cognitive radio technology, there still exist some problems and challenges. For example, further research is needed on how to improve the accuracy of spectrum sensing and how to achieve dynamic spectrum access. In addition, the development and research of the integration of cognitive radio and existing wireless communication systems are also an important research direction.

In order to solve the above challenges, lots of efforts have been made in the CR research area, including basic theory, radio frequency (RF) front end, spectrum sensing, adaptive modulation wave technology, smart antenna, etc. Furthermore, studies have also explored multiple input multiple output (MIMO) and orthogonal frequency division multiplexing (OFDM) technologies to improve the efficiency and reliability.

As a radio communication system based on artificial intelligence technology, CR can automatically analyze different frequencies through the cognition of radio signals and realize intelligent switching and automatic modulation of radio signals. In this light, it has been widely used in many fields, including aviation, automobiles, smart homes, Internet of Things, and its potential application scenarios are very wide, which are mainly discussed in this paper.

The basic principles of cognitive radio technology can be summarized as follows: first, the system needs to use certain sensors and computer hardware to collect and process radio signals. Then, it also has to process and convert the signal data collected by sensors and computer hardware in order to achieve automatic recognition and analysis of the signal. Finally, automatic selections of the system involve the corresponding modulation method, demodulation method, frequency, and so on based on the recognized signal type.

In the application of cognitive radio technology, different frequencies and signals need to be identified and analyzed, which requires special processing and conversion of radio signals. This processing and conversion require the use of special sensors and computer hardware, so the cost of cognitive radio technology is high. In addition, cognitive radio technology needs to comply with specific laws and regulations in the application process, which also requires system manufacturers and users to comply with relevant regulations and standards to ensure the stability and safety of the system.

The applications of CR technology in civil aviation, which are discussed in this paper, improve spectral efficiency, communication reliability, and flexibility. CR can perceive and monitor spectrum usage and identify idle frequency bands or shareable spectrum resources. Firstly, the available spectrum can be managed and planed effectively, ensuring that spectrum resources between different communication systems have no interference with each other. Secondly, the available spectrum can be dynamically allocated to different communication systems and users with real-time requirements. Finally, spectrum sharing among multiple communication systems can also be achieved, thereby improving spectrum utilization efficiency and system capacity.

In the field of civil aviation, the application of cognitive radio technology will be widely promoted. For example, airports, airlines, and navigation equipment manufacturers need to monitor and manage radio signals to ensure safe communication and flight operations. This kind of monitoring and management needs to identify and analyze radio signals, and cognitive radio technology is able to meet this demand.

It can be seen that CR technology can play a crucial role in the civil aviation field by enabling intelligent monitoring and management of radio signals. It will significantly enhance the safety and efficiency of flight operations, providing a reliable guarantee for smooth and secure flights. Moreover, cognitive radio technology introduces innovative ideas and technical solutions for intelligent monitoring and management of wireless electrical signals, paving the way for advanced advancements in this domain.

The organization of this article is as follows: Section 2 provides an overview of the fundamentals of cognitive radio technology, including the architecture of a cognitive radio network. In Section 3, we present an overview of the existing enabling techniques for CR communications. Section 4 comprehensively analyzes the current research in cognitive radio. The applications and trends of cognitive radio networks are discussed in Section 5. Section 6 highlights the specific challenges that cognitive radio faces in today’s context. The application of cognitive radio in the field of civil aviation is discussed in Section 7. Finally, Section 8 concludes this paper, summarizing the key findings and contributions.

## 2. Fundamentals

### 2.1. Main Characteristics of Cognitive Radio

Wireless networks face a significant challenge of limited energy and bandwidth, which restricts the quality of service and channel capacity. To address this issue, researchers are exploring new communication and networking paradigms that can intelligently and efficiently utilize these scarce resources. Cognitive radio is a crucial technology that can enable future communications and networking to utilize network resources in a flexible and efficient manner. Different from traditional communication approaches, CR can adapt the operating parameters such as transmission power, frequency, and modulation type according to the changes in the surrounding radio environment [1]. Cognitive capability is the unique characteristic that allows CR devices to acquire necessary information from the radio environment, such as the transmitted waveform, RF spectrum, communication network type, geographic information, locally available resources and services, user needs, security policy, and so on. Once CR devices gather the needed information, it can dynamically adjust the transmission parameters based on the sensed environment changes to achieve optimal performance [2]. The authors of [1,2] considered CR as an opportunistic or dynamic use of existing bands that addresses the analysis of radio environment and estimation of channel state.

### 2.2. Functions of Cognitive Radio 

Cognitive radio operates on a typical duty cycle that involves detecting spectrum idle space, selecting optimal frequency bands, coordinating spectrum access with other users, and vacating the frequency when a primary user (PU) appears. To support this cognitive cycle, the spectrum sensing, management, and sharing are utilized.

Spectrum sensing allows CR to detect spectrum idle space, which is an unused frequency band that can be utilized by secondary users (SUs). When PUs start using the licensed spectrum again, CR can detect their activity through sensing and vacate the frequency to avoid harmful interference.

**Figure 1:**
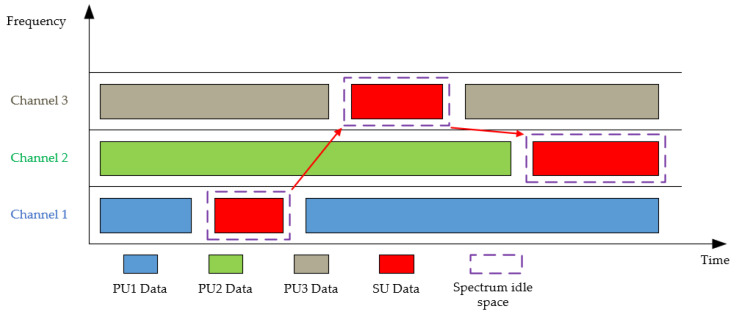
Functional diagram of CR.

After detecting the spectrum idle space, the spectrum management function enables SUs to choose the best frequency band and hop among multiple bands to meet various Quality of Service (QoS) requirements [3]. For example, when a PU reclaims their frequency band, the SU can direct their transmission to other available frequencies, based on factors such as noise and interference levels, path loss, channel error rate, and holding time. A brief process of CR is shown in Figure 1.

Effective spectrum allocation and sharing mechanisms are crucial in dynamic spectrum access to achieve high spectrum efficiency. When SUs coexist with PUs in a licensed band, the interference level due to secondary spectrum usage should be limited by a certain threshold since PUs own the spectrum rights. When multiple SUs share a frequency band, their access should be coordinated to avoid collisions and interference.

### 2.3. Infrastructure of Cognitive Radio Network

Cognitive radio technologies enable SUs to utilize temporally unused licensed spectrum bands owned by PUs. In a CR network architecture, there are both primary and secondary networks. The secondary network comprises a set of SUs with or without a secondary base station. These users can access the licensed spectrum only when it is not occupied by a PU. The opportunistic spectrum access of SUs is coordinated by a secondary base station, which serves as a hub of the secondary network and is equipped with CR functions. If several secondary networks share a common spectrum band, a central network entity, known as a spectrum broker [4], can coordinate their spectrum usage for efficient and fair spectrum sharing.

The primary network consists of a set of PUs and one or more primary base stations. PUs are authorized to use certain licensed spectrum bands under the coordination of primary base stations. Their transmission should not be interfered with by secondary networks. PUs and primary base stations are generally not equipped with CR functions. Therefore, if a secondary network shares a licensed spectrum band with a primary network, the secondary network needs to immediately detect the presence of a PU and direct its transmission to another available band to avoid interfering with primary transmission.

Cognitive communications, through the sensing, detecting, and monitoring of the surrounding RF environment, increase spectrum efficiency and support higher bandwidth service. Moreover, the real-time autonomous decisions for efficient spectrum sharing reduce the burdens of centralized spectrum management. Therefore, CRs can be employed in many applications.

## 3. Techniques

### 3.1. Spectrum Sensing Techniques

Spectrum sensing is a crucial mechanism for obtaining information about the occupancy of the primary spectrum. It aims to identify spectral gaps in different domains such as time, frequency, space, polarization, and angular domains. To achieve this, the secondary transmitter must have an RF chain equipped with sensing capability, which enables it to determine the presence or absence of PUs using various signal processing techniques. Some of the commonly used spectrum sensing techniques include energy detection (ED), feature detection, matched filter-based detection, autocorrelation-based detection, covariance-based detection, and eigenvalue-based detection [5,6,7].

### 3.2. Spectrum Management

Dynamic spectrum management is a technique that involves the efficient allocation and reallocation of spectrum resources to optimize their utilization while preventing interference. Cognitive radio devices are capable of dynamically assigning and reassigning spectrum resources based on their availability and the requirements of users, while taking into consideration the existence of PUs. This approach can enhance spectrum efficiency and ensure effective spectrum sharing between primary and SUs [8].

### 3.3. Spectrum Sharing

This technique involves sharing spectrum resources among multiple users, including both primary and SUs. Cognitive radio devices can share spectrum resources by using techniques such as time division multiple access (TDMA), frequency division multiple access (FDMA), and code division multiple access (CDMA) [9,10].

### 3.4. Resource Allocation

The technique of resource allocation involves the efficient management of the limited radio resources, including power, bandwidth, and time, among different users to enhance the overall system performance. Resource allocation can be performed centrally or in a distributed manner. With the use of intelligent techniques, cognitive radio can optimize spectrum usage and achieve efficient resource allocation, resulting in improved system performance [11,12].

### 3.5. Adaptive Modulation and Coding

Adaptive modulation and coding are a technique used to optimize performance and minimize interference by adapting modulation and coding schemes. Cognitive radio devices are capable of adjusting their transmission parameters, such as modulation and coding, based on the quality of the channel and the needs of the users. By employing adaptive modulation and coding techniques, cognitive radio devices can optimize the use of the available spectrum and improve overall system performance.

CR technology is applied to manage radio signals and provide relevant information on communication protocols and security in cognitive radio networks [13,14], which can be adopted to ensure the safety of communication and flight operations in civil aviation. Ref. [15] provided insights into the practical applications and deployments of cognitive networks, including the potential usage in civil aviation for improved communication and operations. The authors of [16,17] discussed the future research directions and challenges in CR technology, which may provide potential applications in the field of civil aviation. However, the security of CR also deserves attention. Ref. [18] focused on security and privacy protocols specifically designed for cognitive wireless sensor networks.

## 4. Research Status of Cognitive Radio

In [19], the researchers presented a compact, low-cost dual-port filtered antenna system for interleaved cognitive radio applications. The filtered antenna system consists of a frequency bandwidth reconfigurable narrowband filtering antenna (RNF) and a wideband filtering antenna (WF), both of which have a sharp suppression in the stopband. RNF switches between two narrowbands, including WiMAX, 5G, and WLAN frequency bands. The working frequency of WF covers a certain range, which is sufficient to cover the two frequency bands of RNF. The proposed filter produces a flat response in the passband, with two sharp transmission zeros at the stopband.

A two-unit MIMO antenna for cognitive radio MIMO applications was proposed in [20] to avoid the complexity involved in reconfigurable antennas and improve spectral efficiency. The proposed MIMO antenna possesses salient features, such as polarization diversity and performing a maximum of four communication tasks when all the white spaces are detected.

With the discussion of parametric analysis and design and results of the frequency and pattern reconfigurable antenna (FPRA), a new compact reconfigurable antenna was presented in [21]. FPRA has a circular patch with a circular stub on top and a slotted defective ground structure (DGS) on the bottom. In frequency reconstruction mode, FPRA can switch between ultra-wide bandwidth (UWB, 3.05~11.45 GHz), multiband (4 GHz, 6.5 GHz, and 9.2 GHz), and narrowband (7.1 GHz) modes. In UWB and narrowband operations, the radiation pattern is almost omnidirectional, while in broadband situations, the pattern can be reconfigured without affecting the operating frequency band. The reflection coefficient of FPRA is less than −15 dB, and the gain is greater than 2 dBi. The proposed FPRA was processed and validated. The almost constant group delay within the UWB frequency range confirms the excellent time-domain characteristics of FPRA.

A new method to identify unfilled PU spectrum was proposed in [22], which analyzed the collaboration between users using machine learning methods. Learning methods are applied to construct classifiers, appropriate fusion algorithms are selected for the environment under consideration, and efficient out of band sensing is performed. They studied the impact of fading on perceptual performance and found that with the presence of fading, perceptual performance decreases, which is consistent with early research results. From the simulation results, it can be inferred that Weibull fading is superior to all other fading models. Achieving a 1% miss detection probability in the Rayleigh channel yields a false alarm probability of almost 0.8, while achieving the same miss detection probability in the Weibull channel yields a false alarm probability of less than 0.1, which is very beneficial for both indoor and outdoor scenarios. Here, using the time series prediction learning method, a hidden Markov model is used for numerical analysis of the PU channel condition. It is evident that the prediction performance has reached 100% as the result of using the Weibull fading model for a period of 200 ms when compared to the Rayleigh model which achieves only 84.5% accuracy in prediction.

In [23], the author proposed a power allocation method for underlay energy acquisition cognitive radio networks based on supermodel games. An operable market-driven cognitive radio scheme was presented to improve spectrum utilization in radio and television frequency [24].

The early radio navigation equipment can only obtain the single function of the ship’s position at sea by receiving radio signals, but with the development of information technology, the radio navigation system equipment in shipping has been effectively upgraded and updated, better meeting the requirements of modern navigation [25].

Ref. [26] introduced the key technologies of radio applications: wireless spectrum, adaptive transmission, cross layer design. The specific applications of GNU radio integration in mobile communication is also proposed, which are described one by one from high-speed rail and military aspects, so as to highlight the functions of cognitive radio technology and explore its development prospects.

As a conclusion, the research directions and contributions of the latest works mentioned above are shown in Table 1.

## 5. Applications and Trends in Cognitive Radio Networks

**Figure 2:**
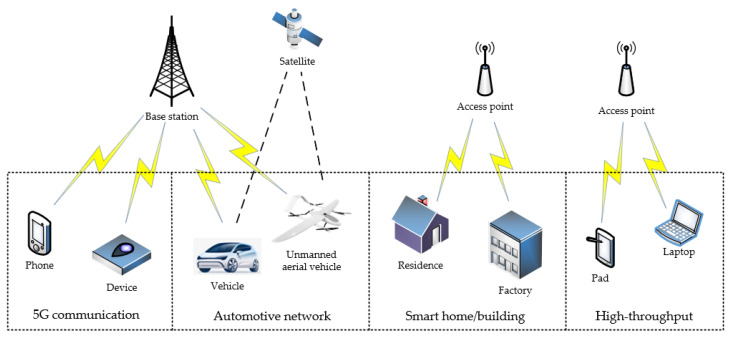
Development directions of CR networks.

With the continuous development of cognitive radio networks (CRNs), people have gradually deepened their understanding of its development direction. At present, the development directions of cognitive radio technology mainly focus on the following aspects (see Figure 2).

### 5.1. 5G Communication System

With the emergence of the 5G era, conventional wireless communication technologies are no longer adequate to meet future demands. Cognitive network technology is increasingly gaining traction as a dominant force in the future development trend. As an emerging network communication technology, cognitive networks will have a significant impact on the future of communication. The development direction of cognitive radio in the context of 5G communication systems is focused on achieving higher data transmission rates, better coverage, and more flexible and diverse communication modes [27].

### 5.2. Mobility and Automotive Networks

The use of multichannel operations in wireless access for vehicular environments (WAVE) has been proposed in the IEEE 1609.4 standard. The WAVE system employs the 75 MHz spectrum in the 5.9 GHz band, with one control channel and six service channels. However, there is still a scarcity of spectrum which poses a challenge for the WAVE system. The application of cognitive radio technology in WAVE has been studied to address this problem [28,29,30]. Some initial work on cognitive radio-enabled vehicular communications has already been conducted. Vehicular wireless sensor networks are becoming a popular network paradigm for collecting monitoring information in urban environments. In this field, CRNs are likely to play a significant role, and protocols for highway safety using CRNs have already been proposed [31,32], although more research is needed in this area.

### 5.3. High-Throughput Applications

Delivering multimedia applications, such as on-demand or live video streaming, audio, and still images, over resource-constrained CRNs is a significant challenge due to their high bandwidth requirements. Refs. [33,34,35] further discuss the advantages of CRNs in these high-throughput application scenarios. Similarly, other CRN applications, including those used in hospitals, vehicular networks, tracking, and surveillance, have varying data densities and require significant bandwidth, low delay, and high bursts of data. However, because SUs in CRNs can access multiple channels whenever necessary and available, CRNs are well suited for bandwidth-intensive applications.

### 5.4. Smart Home or Building Applications

A wide range of indoor applications, such as intelligent buildings, home monitoring systems, factory automation, and personal entertainment, require a dense CRN environment to ensure an adequate Quality of Service (QoS). However, conventional CRNs face significant challenges in achieving reliable communication in indoor areas due to the extremely crowded industrial, scientific, and medical (ISM) bands [36]. To overcome these challenges, CRNs offer a promising solution. By using cognitive radio technology, CRNs can mitigate the limitations of traditional indoor CRN applications, thereby improving their efficiency and effectiveness.

### 5.5. Immediate Monitoring Applications

Immediate monitoring applications, such as traffic monitoring, biodiversity mapping, habitat monitoring, environmental monitoring, crop and livestock monitoring, irrigation, underwater CRNs, vehicle and inventory tracking, disaster relief operations, as well as bridge and tunnel monitoring require reliable and low-latency communication with minimum channel access. For some real-time surveillance applications, even a small delay can be critical, and they therefore require high reliability. However, in multihop CRNs, a delay can occur due to link failure if the channel condition is poor. In contrast, CR nodes can switch to another channel with a better condition if they find one. To increase the channel bandwidth, CRNs can use channel aggregation and multiple channels concurrently, which can be beneficial for real-time surveillance applications [37,38].

### 5.6. Military and Security-Related Applications

Conventional cognitive radio networks find application in numerous military and public security scenarios. Some of these applications include chemical, biological, radiological, and nuclear (CBRN) attack detection and investigation, command and control operations, gathering information for battle damage evaluation, battlefield surveillance, intelligence assistance, targeting, and more. In hostile regions, adversaries may employ jamming signals to interfere with radio communication channels. In such situations, CRNs can switch to different frequency bands to avoid the band with the jamming signal. Furthermore, certain military applications require large bandwidths, minimal channel access, and low communication delays, making CRNs an optimal choice for these purposes.

Authors have achieved some research results in cognitive wireless networks and cognitive vehicular networks [39,40]. There are also some novel research directions in applying cognitive radio networks in civil aviation such as air–ground communication, cognitive radar, etc.

In general, the future development direction of cognitive radio technology is mainly to achieve more efficient communication and integration with other communication technologies.

The future development direction of cognitive radio mainly includes the following aspects:

Firstly, incorporating artificial intelligence (AI) technology into cognitive radio enables signal adaptation, autonomous sensing, and intelligent modulation, leading to enhanced system performance. Furthermore, integrating predictive, analytical, and reasoning capabilities into the cognitive radio system can augment its intelligence and efficiency.

Secondly, high-speed transmission is crucial. With the introduction and continuous upgrading of various new wireless technologies, the demand for higher transmission rates has surged. Consequently, cognitive radio technology must enable faster data transmission rates while ensuring data accuracy and reliability [41].

Thirdly, security is a crucial consideration in cognitive radio systems as the number of wireless devices increases. To ensure the stability and reliability of the system, more attention and research are required to strengthen the security protection of cognitive radio systems in the future.

## 6. Challenges Faced by Cognitive Radio

Cognitive radio refers to the use of radio technology to obtain, process, and interpret the information transmitted and processed by electronic equipment and communication systems.

The challenges faced by cognitive radio are as follows:

### 6.1. Limitations on Frequency Range

The limitation of the cognitive radio frequency range is that human cognition and perception are limited by the radio frequency range. In modern communication technology, the use of radio frequencies has become an unavoidable problem, and these frequency ranges have a profound impact on human perception and cognition.

The frequency spectrum of radio waves has a wide range from 3 kHz to 300 GHz, which includes many frequencies required by modern communication technologies, such as Wi-Fi, Bluetooth, mobile data, satellite communication, etc. However, these frequencies impose some limitations on human perception and cognition [42,43].

Firstly, the frequency of radio waves may result in different frequency ranges received by devices exposed to radio waves, which may affect their perception and cognition. For example, the frequency range of Wi-Fi radio waves is between 2.4 GHz and 2.45 GHz, but different Wi-Fi devices may have different frequency ranges, which may lead to different wireless access points (APs) connecting to the network, thereby affecting the perception and cognition of the devices.

Secondly, the frequency of radio waves may also affect the perception and cognition of the eyes and brain. The shorter the wavelength of a radio wave, the better its penetration ability. This means that high-frequency radio waves can penetrate fog and obstacles, while shortwave radio waves are more easily received by radar antennas. However, the wavelength of high-frequency radio waves is relatively short, so the human eye’s perception and cognition of shortwave radio waves may be affected, which may lead to high-frequency radio wave devices being considered ineffective.

Finally, the frequency of radio waves may also affect human cognition and culture. In some cultures, radio waves are considered an important cultural element, such as the radio and television frequency bands in China and some radio frequency bands in other countries, such as the 1.1 GHz and 1.8 GHz frequency bands in the UK, which are considered to have cultural significance. The use of these frequency bands may affect people’s cognition and culture.

Therefore, as human cognition and perception ability are limited by the radio frequency range and different radio frequencies may affect human perception and cognition, so different frequency ranges are required.

### 6.2. Increase in Complexity

Cognitive radio technology, also known as cognitive communication, is a cross field research area involving radio technology and artificial intelligence technology. The development and widespread application of this technology have led to a deeper understanding of communication and computing methods, while also increasing the complexity of radio technology. The development of cognitive radio technology is inseparable from the support of artificial intelligence technology. The application of artificial intelligence technology makes the training of algorithms, data structures, and models more efficient, and also makes the process of machine learning applied to cognitive radio more complex [44,45].

There are papers and patents involving the applications of CR technology in different fields. For example, Ref. [46] proposed a dual-user relay protocol based on energy harvesting and cognitive radio technology, which achieved energy efficiency via relay communication. Ref. [47] compared the application potential and performance differences in different CR technologies in the FM broadcast frequency band. Ref. [48] proposed a novel channel allocation method to improve spectrum utilization efficiency with an OFDM-based CR technology. Ref. [49] introduced a CR technology utilizing a multiphase downconverter to efficiently receive multiple signals. A research report from Ain Shams University had reported a CR technology utilizing major L-band ranging devices for aviation communication [50]. These related works provided valuable knowledge and technical solutions for the academic and industrial communities.

The development of cognitive radio has also increased the complexity of radio technology. The development and improvement of radio technology changes with the change in cognitive radio technology. For example, antenna array, modulation mode, demodulation mode, signal power transmission, etc. are closely related to cognitive radio technology [51].

Finally, cognitive radio technology also needs to identify and understand the signal, which requires compression, quantization, noise removal, reverberation removal, shock removal, and other operations on the signal. These operations require in-depth research and analysis of the signal in order to extract useful information [52].

To sum up, the development of cognitive radio technology enables people to have a deeper understanding of communication and computing and also increases the complexity of radio technology. The development of cognitive radio technology makes the cross field research between computer and radio technology more complex and efficient [53,54,55].

The research of cognitive radio technology needs the cross knowledge of computers, artificial intelligence, radio technology, and other fields. The cross research in these fields not only increases the complexity of technology but also promotes the progress and application of technology [56,57].

### 6.3. Efficient Data Storage and Processing

In cognitive radio, data storage and processing are very important. Traditional data storage requires a lot of disk space and professional equipment support, which is unrealistic for cognitive radio applications with high real-time requirements. Cognitive radio data storage and processing use efficient algorithms and hardware support, making data storage and processing more efficient [58].

The high efficiency of cognitive radio data storage is because it uses distributed storage technology to store data on multiple nodes, so as to achieve efficient data storage and processing. Compared with traditional data storage methods, cognitive radio data storage can access and process data faster, while reducing the number and complexity of storage devices [59].

Cognitive radio data storage also uses adaptive algorithms, which can automatically select suitable storage nodes and storage methods according to the real-time needs, size, and mode of data and other factors. This algorithm can reduce the workload of storage devices and improve their response speed and efficiency [60].

In conclusion, the efficiency of cognitive radio data storage is the key reason for its wide application in the field of radio communication. By using distributed storage technology, efficient algorithms and hardware support and other technical means, cognitive radio data storage can efficiently store and process data, providing strong technical support for the development and application of cognitive radio communication technology [61,62,63].

### 6.4. Security Issues

Cognitive radio is a new radio communication technology, which can use artificial intelligence and machine learning algorithms to design and generate radio signals, so as to achieve extensive radio coverage and low-power wireless sensor networks. In cognitive radio technology, information and data are encoded as radio signals and transmitted through radio waves, which can be received and processed by different devices [64].

However, the security of cognitive radio also deserves attention. With more and more devices using cognitive radio technology, how to handle and protect the security of these devices becomes particularly important. Among the security issues of cognitive radio, the following are the main issues:

- Interference and deception.

The transmission and reception of cognitive radio signals require low power consumption and short-distance communication, so they are vulnerable to interference and deception. These interferences and deceptions can be caused by the visibility, interference, or deception of other devices, networks, or technologies, as well as specific frequency noise and other interferences. Therefore, when using cognitive radio, it is necessary to ensure that the equipment and network are secure to avoid interference and deception [65,66,67].

- Data integrity.

Cognitive radio devices can receive and transmit data, which may be damaged or tampered with. In order to protect data integrity, data encryption and integrity verification must be implemented. This will ensure that only the correct devices can access and transmit data and prevent data from being tampered with [68,69].

- Unknown risks.

The use of cognitive radio technology involves unknown risks and vulnerabilities, which may pose a threat to the security of equipment. Therefore, it is necessary to test the security of devices and networks and identify and fix vulnerabilities and potential security risks, in order to ensure the security of devices [70].

- Lack of standardization.

At present, cognitive radio technology lacks standardization. This means that devices from different manufacturers may have different safety and functionality, and there is a lack of unified testing and evaluation standards. This may pose a threat to the security and stability of the devices, as well as prevent them from communicating with the network or working properly [71].

Cognitive radio technology has great potential and broad application prospects, but security issues must be addressed and solved to ensure the safety and correct use of equipment. This includes implementing standardization, testing and verifying the integrity and safety of equipment, as well as developing unified safety standards and testing processes. This will ensure the security of devices and networks and enable a wider range of cognitive radio applications [72,73].

### 6.5. Standardization Issues

CR standardization is the core issue of the current technical field. Standardization organizations and relevant research units are working closely together to develop unified standards and testing methods to secure the rapidly developing fields of CR technology. In particular, Ref. [74] pointed out that cognitive radio technology needs to be standardized in multiple aspects, including spectrum management, channel selection, interference detection and management, security, etc. Meanwhile, due to the complexity and diversity of CR technology, the development of standards also should fully consider the differences and special demands of application scenarios.

Cognitive radio is a method of designing, constructing, and testing radio systems using artificial intelligence and machine learning methods. In cognitive radio, the acquisition, processing, and interpretation of signals rely on computer vision and natural language processing technologies. These technologies can enable artificial intelligence systems to recognize, analyze, and interpret human language and signals, so as to better complete the task of cognitive radio [75,76].

Due to the complexity and innovation of cognitive radio systems, standardization is particularly important to ensure that all systems can work in the same environment and meet the needs of users.

At present, the standardization of cognitive radio involves many aspects, such as the layout of radio channels, communication protocols, signal processing algorithms, equipment design, and so on. Although various standardization organizations have already carried out some work, there are still many challenges, such as: different standardization organizations may have different standard definitions, standard testing methods, standard implementation methods, etc., which may lead to inconsistencies between different systems. In addition, the research and development of cognitive radio need a lot of resources, and the workload of standardization organizations will increase accordingly, so more cooperation and support are needed [77].

Standardization organizations and relevant research units need to work closely together to develop unified standards and test methods to ensure that all systems can work in the same environment, meet user needs, and promote the development of cognitive radio technology. It is also necessary to strengthen relevant research results and experimental work to better guide the practice of standardization work. Only in this way can we truly realize the popularization and development of cognitive radio technology and create more communication and cognitive capabilities for humankind [78,79].

### 6.6. Environmental Adaptability Issues

Cognitive radio is a new technology, which involves the conversion of radio signals into cognitive signals so that computers and humans can recognize and understand these signals. This technology has applications in multiple fields, such as medical, military, transportation, and communication. The problem of environmental adaptability of cognitive radio refers to how humans recognize and interpret radio signals in the environment [80].

Cognitive radio technology requires powerful computers and complex algorithms to analyze and understand radio signals. These computers require sufficient memory to process a large amount of data and high-speed memory to store the parsing results. Algorithms also require strong computing power to process large amounts of data in order to convert complex radio signals into cognitive signals [81].

The problem of environmental adaptability of cognitive radio is related to human cognitive processes, and it needs to consider how the human brain processes information. When analyzing radio signals, it is necessary to consider factors such as frequency, strength, and phase of the radio signal in order to convert this information into cognitive information. It is necessary to consider how different human brains process information differences in order to achieve consistency in analytical results among different individuals [82].

The environmental adaptability of cognitive radio needs to consider complex radio signals, which requires strong computing power of analytical algorithms and powerful processing power of computers to process large amounts of data. Humans need to adapt to different radio signals in the cognitive radio environment in order to convert this information into cognitive information and different signal sources are needed in the environment so that different people have different results of analytic signals [83].

The environmental adaptability of cognitive radio needs to consider how the human brain processes information, and different signal sources and resolution algorithms are needed to achieve consistency of resolution results between different human individuals and switch signal sources in different environments [84].

In a word, cognitive radio’s environmental adaptability needs to consider complex radio signals, requires humans to adapt to different radio signal sources and resolution algorithms, and requires switching signal sources in different environments. The application of this technology will bring many changes to humanity, including applications in medical, military, transportation, and communication fields, while also bringing more efficient communication and more accurate diagnosis and treatment to humanity [85].

## 7. Applications of Cognitive Radio in Civil Aviation

### 7.1. Applications in Civil Aviation

In civil aviation, with proper definition and working principles, CR technology can be applied to monitor and manage radio signals, ensuring the safety of communication and flight operations [86].

**Figure 3:**
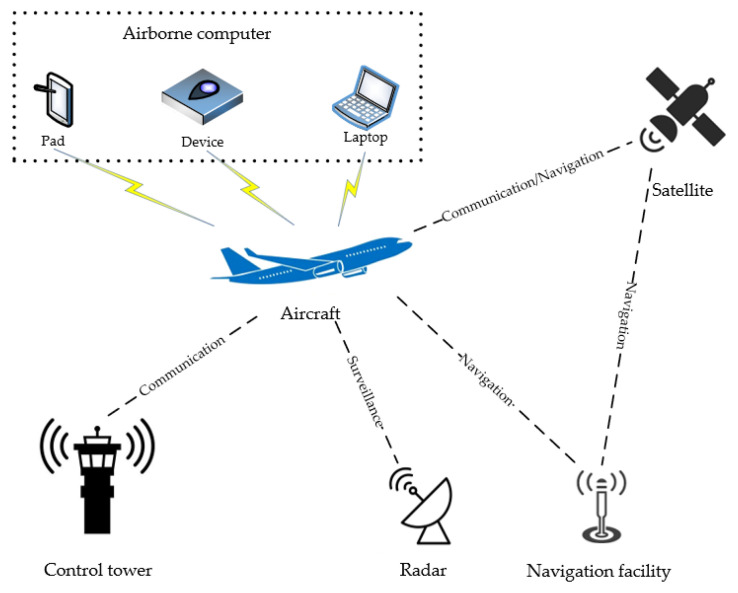
Application scenarios of CR in civil aviation.

The increase in air traffic leads to the increasing use of radio spectrum for aeronautical communications in civil aviation, which is expected to be more and more congested. Considering the advantages of cognitive radio in resource-constrained situations, researchers have begun to discuss the applications of cognitive radio in civil aviation such as air–ground communication, air–air communication, unmanned aerial systems, etc. [87]. Furthermore, cognitive radio technology, along with other radio technologies, can be widely used in the following aspects in Figure 3.

#### 7.1.1. Communication System

The application of CR in civil aviation communication systems refers to the use of new radio technologies and equipment to make it easier and safer for passengers and crew to use communication equipment, including mobile phones, laptops, e-books, etc. In the civil aviation communication system, the application of CR can make flight communication more efficient, safe, and reliable [88].

With the continuous development of modern communication technology, people’s understanding of radio is also constantly improving. Radio technology plays an increasingly important role in modern communication, with applications including radio beacons, radio measurements, radio reception and transmission, satellite communication, and more. CR technology is based on the improvement of people’s radio awareness and, through the use of new radio technology and equipment, passengers and crew can use communication equipment more easily and safely, including mobile phones, laptops, e-books, etc. [89].

In civil aviation communication systems, the application of CR can improve communication efficiency and security. The flight communication system needs to handle different types and quantities of communication devices, such as mobile phones, laptops, e-books, etc. CR can make these devices easier to manage and recognize, while providing better security [90].

Airport wireless beacons are an important component of airport communication systems. They are used for transmitting and receiving wireless network signals, making it easier for passengers and crew members to connect to the airport wireless network and access various applications and resources during flights. CR can replace traditional radio beacons because it uses higher-frequency radio waves, making it easier to connect to wireless networks while providing better signal-to-noise ratio and stability. A radio direction finding system is a type of radio equipment that uses radio waves of a specific frequency to detect and locate radio equipment. In civil aviation communication systems, CR can replace traditional radio direction finding systems by using high-frequency radio waves to detect and locate various communication devices. CR can better detect and locate handheld radio devices, including mobile phones and other communication devices. The airport wireless communication system is an important component of the flight communication system. CR can be used to connect airport wireless communication systems, making it easier for passengers and crew to access flight communication systems, such as flight information, entertainment systems, and flight communication beacons. CR can also provide safer and more efficient communication, enabling passengers and crew members to more effectively access information and maintain flight communication systems [91,92].

The application of CR in civil aviation communication system is an important part of airport communication system and flight communication systems. CR can make it easier and safer for passengers and crew to use communication equipment, improving the efficiency and safety of airport communication systems and flight communication systems [93].

#### 7.1.2. Satellite Communication

Cognitive radio is a new type of radio technology. Different from traditional analog signal radio, cognitive radio uses digital signal technology for communication, which can automatically identify and adapt to different communication needs, including voice communication, data communication, and video communication, and can communicate in different wireless communication systems, such as satellite communication, mobile communication, and terrestrial radio communication.

In the field of civil aviation, cognitive radio technology is widely used to ensure the safety and smoothness of flights. The radio systems of civil aviation airports and airlines are usually maintained and upgraded by professional radio engineers who need to be trained and certified in cognitive radio technology in order to master the latest technologies and tools and ensure the reliability and safety of the radio system [92].

Cognitive radio technology is applied to flight communication, including voice communication, data communication, and video communication. For example, on a flight, communication between the captain and passengers usually uses satellite phones for real-time communication. Since the aircraft will generate noise in flight, the captain’s voice will be interfered with by the noise. Therefore, the captain needs to use cognitive radio technology to record and confirm the call to ensure the quality and safety of the call.

The radio system at the airport is used to monitor the status, flight speed, and dangerous situations encountered by the aircraft, such as the fuel condition, attitude, and altitude of the aircraft. The airport radio system also needs to use cognitive radio technology for monitoring and communication, so that flight and airport management personnel can understand the flight situation in real time.

The application of cognitive radio technology in the radio systems of civil aviation and airports can improve the reliability and security of communication, ensure the safety and smoothness of flights, and ensure the safety and interests of passengers and airport managers.

#### 7.1.3. Navigation

Cognitive radio is a radio communication technology based on machine learning and cognitive computing. Its goal is to optimize and improve the radio communication system by making use of human cognitive and language abilities to enable radio devices to automatically recognize and receive signals. The application of cognitive radio technology in civil aviation navigation can bring the following advantages:

- Improvement of navigation accuracy.

Traditional GPS navigation uses satellite signals, which are influenced by factors such as the Earth’s curvature and satellite signal attenuation, resulting in navigation errors. Cognitive radio technology uses cognitive computing and machine learning algorithms to identify and correct satellite signals in real time and improve navigation accuracy.

- Reduction of wireless channel interference.

Wireless channels are an important part of communication systems, but they are often affected by interference from other communication signals, which poses a threat to the integrity and accuracy of the signal. Cognitive radio technology can automatically identify and avoid interference and improve the stability and accuracy of radio channels, thus ensuring the accuracy of navigation.

- Improvement of aircraft safety.

During aircraft flight, navigation equipment must ensure flight safety. Traditional navigation methods require manual signal confirmation and operation, which are susceptible to human errors and fatigue. The cognitive radio technology can be used by the crew to confirm the radio signal through simple operation, so as to reduce the occurrence of human errors and improve flight safety [91].

- Improvement of aircraft communication.

A traditional aircraft communication system is mainly composed of a mechanical navigation system and radio navigation system. The mechanical navigation system is vulnerable to bad weather and mechanical failure. The communication of the radio navigation system is vulnerable to signal interference and interruption, resulting in reduced reliability of navigation information. Cognitive radio technology can improve the communication between the aircraft mechanical navigation system and radio navigation system while maintaining the stability of radio signals.

The application of cognitive radio technology in civil aviation navigation can improve navigation accuracy, reduce radio channel interference, improve aircraft safety and improve aircraft communication, and lay a solid foundation for accurate flight, safe flight, and reliable operation of flights.

#### 7.1.4. Airborne Computers

Airborne computers are the core computer systems used by airlines on aircraft. Cognitive radio technology is used to receive signals needed by airborne computers and convert them into formats that can be processed by computers. For example, airlines can use cognitive radio technology to receive data required by airborne computers, such as flight plans, weather data, etc., so that airborne computers can process these data and execute tasks.

Cognitive radio technology plays a vital role in the field of civil aviation. With the development of computer and digital communication technology, cognitive radio technology is constantly updated to meet the needs of flight communication and improve the quality and efficiency of flight communication.

The application of cognitive radio technology in the field of civil aviation involves communication systems, satellite communications, navigation and airborne computers. With the continuous development of technology, cognitive radio technology is becoming more and more important and influential in the field of civil aviation [94].

### 7.2. Development Trend of Cognitive Radio in Civil Aviation

Cognitive radio technology in the field of civil aviation has received extensive attention and research. In the future, this technology will be more widely applied in the field of civil aviation, with the following main development trends:

#### 7.2.1. Higher Security and Reliability

Cognitive radio technology can improve flight safety by identifying and tracking the surrounding signal environment. In the future, cognitive radio technology will adopt more security mechanisms and adaptive algorithms to improve its reliability in response to unexpected situations such as communication interruption.

#### 7.2.2. Multimodal Communication

In the field of civil aviation, an aircraft must communicate with ground control consoles, other flights, weather stations, and other aircraft simultaneously, thus achieving multimodal communication is necessary. Cognitive radio technology will respond to this demand and support multiple communication modes to improve the utilization of the radio spectrum.

#### 7.2.3. Higher Spectrum Utilization

One of the development trends in the future is to improve the spectral efficiency. In the case of scarce spatial spectrum resources, improving the spectral efficiency of cognitive radio technology has become a major goal. Combining OFDM and MIMO technology with cognitive radio technology can achieve more efficient spectrum utilization.

#### 7.2.4. Advanced Algorithms

With the improvement of computing power and processing speed, cognitive radio technology can support more complex and advanced algorithms and models to solve some complex problems that have not been solved in the existing technology. For example, machine learning, artificial intelligence, and other technologies will be widely used in cognitive radio technology to improve its performance and accuracy [95].

#### 7.2.5. Integration with Other Technologies

Cognitive radio technology will be combined with other technologies to support more applications. For example, combined with aviation control center technology, cognitive radio technology can improve the communication efficiency between different flights and provide more secure and reliable technical support for flight scheduling. 

In conclusion, cognitive radio technology will play an important role in the field of civil aviation. With the development of technology and the increase in application scenarios, cognitive radio technology will show a broader and flexible development prospect.

### 7.3. Future Prospects of Cognitive Radio in Civil Aviation

Based on the development trend of cognitive radio technology, we can predict that it will play an important role in the field of civil aviation. First of all, using cognitive radio technology, aircraft communication, navigation, monitoring, control and other systems can achieve autonomous, cooperative, reliable, and efficient operation. Secondly, cognitive radio technology can process the sensing information obtained by the aircraft with high accuracy, efficiency, and speed, so as to realize prediction, control, and intervention operations and improve the performance and safety of the aircraft. At the same time, cognitive radio technology can also effectively reduce congestion, delay, and other problems in the civil aviation field and improve the overall operation efficiency and service quality through intelligent data analysis and optimization. 

In the future, the application of cognitive radio technology in the field of civil aviation will be more popular and widespread. First, with the gradual maturity and improvement of related technologies, the performance of cognitive radio systems will be greatly improved, which can better meet the needs of practical applications. Secondly, the application scope of cognitive radio technology will continue to expand, covering more fields and scenarios, including control and command, airport operation, aircraft maintenance, etc. In the future, more application innovations may emerge, continuously improving the overall operational efficiency and safety of the civil aviation system.

However, the application of cognitive radio technology in the field of civil aviation still faces some challenges and problems. First, with the continuous development of civil aviation and the continuous expansion of flight control scale, the channel resource utilization and spectrum coexistence of cognitive radio technology will become more and more complex. Secondly, the application of cognitive radio technology needs to optimize and coordinate the resource allocation and scheduling of different systems and needs to solve many technical problems such as information security, equipment standards, mutual interference, etc. 

Therefore, the future direction of development should be to pay more attention to the research and innovation of new technologies and develop more efficient, accurate, and intelligent cognitive radio systems to meet the needs and challenges of flight network operation. While achieving functional upgrades, we also need to develop unified standards and specifications, promote technical coordination and information sharing in the field of civil aviation, integrate and optimize civil aviation resources, and achieve more efficient, safe, and reliable operation of the civil aviation network.

## 8. Conclusions

In the future development of cognitive radio, there are many trends and prospects. First of all, research in cognitive radio will pay more attention to the development of intelligence and strive to continuously improve its intelligence level, so that it can more accurately perceive the radio environment and more accurately adjust the use of radio resources. Secondly, research in cognitive radio will seek better technology combinations to improve the utilization and efficiency of its radio spectrum.

In addition, the future development direction of cognitive radio will also involve its application in the field of civil aviation. The civil aviation industry, as a crucial field for air traffic safety, has high requirements for the safety and reliability of radio usage. The future development direction of cognitive radio will be closely combined with the civil aviation field to improve the reliability and safety level of its communication and navigation.

Finally, cognitive radio will continue to face various challenges and problems, such as insufficient spectrum resources, interference, etc. Therefore, in the future development of cognitive radio, it is necessary to constantly strengthen technical research and improve the technical level to meet these challenges and problems. The future development of cognitive radio will pay more attention to technological innovation and intelligent development and use radio in a more efficient, safer, and more reliable way by combining various advanced technologies. At the same time, the future development of cognitive radio also needs to strengthen the solution of existing problems and constantly solve new problems to promote its continuous development and growth.

## Figures and Tables

**Table 1:** The latest research directions and contributions.

Reference	Research Field	Method	Contribution
Ref. [19]	Filtered antenna system	Dual-port filtered antenna	Flat response in the passband with two sharp transmission zeros at the stopband
Ref. [20]	MIMO antenna system	Two-element MIMO antenna	Possesses salient features, e.g., polarization diversity
Ref. [21]	FPRA for UWB and CR application	A new compact reconfigurable antenna	Suitable for UWB and CR application
Ref. [22]	Idle spectrum prediction	Time series forecasting learning method	Promotion in the prediction performance
Ref. [23]	Power allocation in CR	Supermodel game power allocation method	Energy acquisition allocation scheme
Ref. [24]	CR application	A novel market-driven CR scheme	Improves spectrum utilization in radio and television frequency
Ref. [25]	CR communication and networking	CR schemes in maritime navigation	Proves the possibility of CR in maritime navigation
Ref. [26]	GNU radio and CR application	GNU radio integration in mobile communication	Highlights CR technology and explores its development prospects

## Data Availability

The data used to support the findings of this study are included within the article.
